# Improving CKD Screening and Care in Diabetes Using Clinical Decision Support in a Large Health Care System

**DOI:** 10.34067/KID.0000000829

**Published:** 2025-04-24

**Authors:** Ken J. Park, Michalah K. Tandy, Shaun Flerchinger, Kathryn J. Glassberg, Frank Y. Chen, Eric S. Albright, Lisa J. Nakashimada

**Affiliations:** 1 Department of Nephrology, Kaiser Permanente Northwest, Portland, Oregon; 2 Department of Predictive Analytics, Kaiser Permanente Northwest, Portland, Oregon; 3 Department of Internal Medicine, Kaiser Permanente Northwest, Portland, Oregon; 4 Department of Hospital Medicine, Kaiser Permanente Northwest, Portland, Oregon; 5 Department of Endocrinology, Kaiser Permanente Northwest, Portland, Oregon; 6 Department of Quality, Kaiser Permanente Northwest, Portland, Oregon

**Keywords:** ACE inhibitors, albuminuria, CKD, diabetes mellitus, SGLT2

## Abstract

**Key Points:**

- Clinical decision support tools in a large health system increased CKD screening in diabetics from 35% to 72%.
- Clinical decision support tools resulted in mixed results in CKD quality but was associated with increase in sodium-glucose cotransporter 2 inhibitor use.

**Background:**

Guidelines recommend screening for CKD in patients with diabetes with annual urinary albumin-creatinine ratio (ACR) and serum creatinine (Scr). However, screening rates were low in Kaiser Permanente Northwest, a large integrated health care system. We implemented a quality improvement project using clinical decision support (CDS) tools to increase ACR and Scr testing. We examined whether increased CKD screening resulted in improvement in CKD quality metrics, specifically angiotensin-converting enzyme inhibitors/angiotensin receptor blockers and sodium-glucose cotransporter 2 inhibitor (SGLT2i) use.

**Methods:**

In May 2022, we implemented CDS tools to increase ACR/Scr testing consisting of automated laboratory ordering, best practice alerts (BPAs), and automated laboratory reminders to patients through letters, texts, and phone calls in tandem with provider education on best practice recommendations for CKD. A SGLT2i BPA targeting patients with type 2 diabetes with ACR ≥300 mg/g and eGFR ≥30 ml/min was rolled out in June 2022 and expanded to include patients with eGFR ≥60 ml/min regardless of CKD diagnosis in February 2023. Trends were reviewed monthly using statistical process control charts and changes in slope using segmented regression analysis.

**Results:**

After 3 years, ACR/Scr testing conducted within 1 year increased from 35% to 72%. Angiotensin-converting enzyme inhibitor/angiotensin receptor blockers use increased slightly from 74% to 76%, but nephrology comanagement for high-risk CKD patients remained unchanged at 53%. The rate of SGLT2i use steadily increased by 0.6% each month up until 6 months after introduction of the BPA, after which the rate increased to 1.7%. Among patients not comanaged with nephrology, the adjusted rate of increase was 7% higher in the BPA group compared with patients with CKD in the non-BPA group.

**Conclusions:**

Our study shows that the use of CDS tools improve CKD screening in patients with diabetes but with mixed results in CKD quality metrics.

## Visual Abstract

**Figure:**
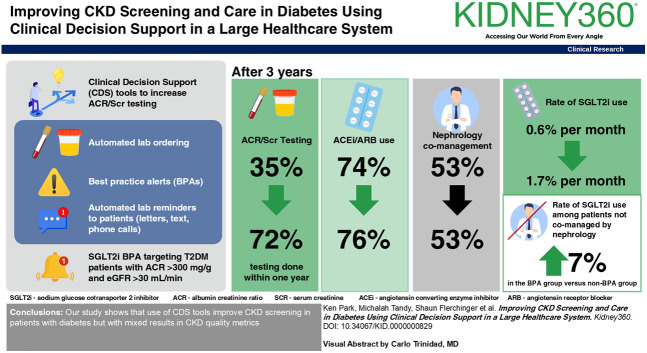

## Introduction

### Background/Available Knowledge

Diabetes is the leading cause of CKD, affecting 20%–40% of patients.^1^ Despite its high prevalence, CKD in diabetes often goes unrecognized. Studies show low CKD screening rates, primarily for urine albumin-creatinine ratio (ACR), at 10%–40%.^2–4^ In addition, angiotensin-converting enzyme inhibitors (ACEi) or angiotensin receptor blockers (ARBs) and sodium-glucose cotransporter 2 inhibitors (SGLT2i) remain underprescribed despite strong evidence that they slow CKD progression and reduce major adverse cardiovascular events (MACEs).^5–7^ Recent analyses suggest that population-wide screening for CKD, including in patients with diabetes, could be cost effective, and annual screening is recommended by several major organizations.^8–10^ Studies have shown that ACR testing was associated with higher rates of receiving ACEi/ARB or SGLT2i, which can lead to earlier initiation of effective therapies that can delay the need for dialysis and reduce MACE.^11–13^ Clinical decision support (CDS) tools can provide clinicians with individualized patient recommendations and have been used in diabetes, atherosclerotic cardiovascular disease (ASCVD), and congestive heart failure (CHF) to improve quality of care.^14–16^ However, the results of trials using CDS tools in CKD have been mixed.^17–20^

### Problem Description, Rationale, and Specific Aims

About 39% of patients with diabetes completed annual ACR and serum creatinine (Scr) testing in Kaiser Permanente Northwest (KPNW). When we interviewed primary care providers, common themes were lack of awareness of recommendation for annual ACR testing, lack of time to assess if a patient had ACR or Scr checked, or the misconception that ACR was automatically ordered. We implemented a quality improvement (QI) project with the primary goal of increasing the percentage of patients with diabetes who had a Scr and ACR measured within the past year to 70% 1 year after implementation of CDS tools, which represented the average rate across all Kaiser regions. Our secondary goals were to increase prescribing of ACEi/ARBs, SGLT2is, and nephrology referrals of patients with high-risk CKD through a combination of best practice recommendations and provider education.

## Methods

### Context

Our QI project targeted patients enrolled in KPNW, a large integrated health care system serving the Portland, Oregon metro area and southwest Washington. KPNW serves around 616,000 members and is comprised of 1300 providers including physicians, physician assistants, and nurse practitioners in 60 medical offices and 4 medical centers. Health information, including demographics, clinical encounters, medications, and laboratory results, are stored in a comprehensive electronic health record (EHR; Epic, Verona, WI). Providers are supported by informatics tools integrated into the EHR, including disease registries, automated laboratory ordering based on registry membership, and CDS tools providing best practice recommendations.

### Intervention

We targeted patients between the ages of 18 and 85 years who were automatically enrolled in a diabetes registry if they had a problem list diagnosis of type 1 or 2 diabetes or associated complications (International Classification of Diseases, Tenth Revision code E10.xx). Patients with ESKD, on hospice or palliative care, <1-year enrollment, living in long-term care facilities, older than 66 with frailty and advanced illness, or older than 81 with frailty were excluded. The first step consisted of education of primary care providers, including nurse practitioners and physician assistants, on recommendations for annual ACR and eGFR testing in patients with diabetes, indications for nephrology referral, and management of CKD including indications for SGLT2i and ACEi/ARBs. Education consisted of emailed handouts, one 20-minute virtual session at 15 primary care clinics given by nephrologists, and a primary care grand rounds in the 6-month period before implementation of second step.

The second step consisted of auto-lab ordering of Scr and/or ACR in patients who had not had either laboratory resulted within the past year in which these laboratory results were drawn automatically. Patients received automated reminders every 6 months through letters, text messages, phone calls, and a printed handout at any in-person visit in primary and specialty care (Supplemental Figure 1) directing them to the laboratory, with results sent to the primary care provider.

The third step consisted of best practice recommendations for ACR/Scr testing, ACEi/ARB use, and/or SGLT2i use (Supplemental Figure 1). These recommendations were in a printed handout given to primary care providers before clinic. ACEi/ARB use was recommended in patients not currently prescribed one with most recent ACR ≥30 mg/g within 1 year. SGLT2i use was recommended in patients with type 2 diabetes (DM2) with a problem list diagnosis of CKD and most recent eGFR ≥30 ml/min and ACR ≥300 mg/g within the past year. Providers would also see a best practice alert (BPA) in the EHR recommending starting a SGLT2i (Supplemental Figure 1). Although our guidelines recommended SGLT2i use in patients with DM2 and CKD with eGFR ≥30 ml/min regardless of albuminuria, we chose to target this group based on studies showing a lower number needed to treat to prevent renal events and to minimize provider alert fatigue.^21,22^ Patients with most recent Scr >2.5 mg/dl, potassium >5 mEq/L, last systolic BP <100 mm Hg, and/or ACEi/ARB allergy were excluded from the ACEi/ARB recommendation, whereas patients with kidney transplant, pregnant, history of Fournier's gangrene, history of diabetic ketoacidosis, diagnosis of foot or leg ulcer within 3 months, or polycystic kidney disease were excluded from the SGLT2i alert.

This project was conceived in August of 2021 and was approved in December 2021 by quality leadership. This project was reviewed with the lead clinician in each primary care clinic and with the laboratory director before implementation. The CDS tools were built by the informatics department and implemented in May 2022. The best practice recommendation for ACEi/ARB had already been in place since 2009, whereas BPA for SGLT2i was rolled out in June 2022. This project was reviewed by the Institutional Review Board of KPNW and deemed not to require oversight as this was a QI project.

### Measures

We collected deidentified data from the diabetes registry on the fifth of each month. The race-based CKD Epidemiology Collaboration equation was used to calculate eGFR before May 2022, after which eGFR was reported using the non–race-based CKD Epidemiology Collaboration equation.^23^ Patients were defined as having a renal indication for ACEi/ARB if their most recent ACR was ≥30 mg/g within the past 5 years. Patients were defined as having a renal indication for SGLT2i if they had DM2 with CKD and last eGFR between 30 and 59 ml/min, regardless of albuminuria level, or eGFR ≥60 ml/min and last ACR ≥300 mg/g within the past 5 years. We defined CKD as having last eGFR <60 ml/min with a problem list diagnosis of CKD (International Classification of Diseases, Tenth Revision code N18.xx), two eGFR's <60 ml/min ≥90 days apart, or most recent ACR ≥300 mg/g within the past 5 years. Patients were defined as having an indication for nephrology comanagement if they had CKD with last eGFR <30 ml/min or most recent ACR ≥1200 mg/g within the past 5 years, which were the local criteria for referral. Patients were defined as having CHF or ASCVD based on problem list diagnosis (Supplemental Table 1). Nephrology comanagement was defined as patients having had a visit, either virtual or in-person, or electronic consult with nephrology within the past year.

We reviewed monthly trends in the percentage of patients completing ACR and Scr testing within a year in a quality committee consisting of several primary care physicians, pharmacists, nephrologists, analysts, and endocrinologists. As balancing measures, we reviewed monthly trends in retinopathy screening, BP control (defined as last BP <140/90 mm Hg), and diabetes control (defined as hemoglobin A1C <8%). Trends in ACEi/ARB prescription and SGLT2i prescription were reviewed quarterly in a subcommittee consisting of an internist, hospitalist, nephrologist, and endocrinologist.

### Study of Intervention, Analyses, and Ethical Considerations

Trends in ACR and eGFR testing, nephrology comanagement, and ACEi/ARB prescribing were examined using statistical process control (SPC) charts using the qicharts2 package in R.^24^ SPC charts are used in QI projects to assess whether an intervention results in an improvement in the desired outcome.^25^ We examined SPC charts for special cause variation, which signifies nonrandom variation, based on previously defined rules.^26^ We also compared trends in ACR and eGFR testing, nephrology comanagement, and ACEi/ARB prescribing at baseline, before CDS rollout, 1 and 2 year after CDS rollout using a chi-squared test for proportions. We defined *P* < 0.05 as statistically significant.

Segmented regression analysis was used to assess trends in SGLT2i prescribing over time in patients with CKD with DM2 and eGFR ≥30 ml/min using the segmented package in R.^27^ We split the patients with CKD into the BPA group (defined as most recent ACR ≥300 mg/g up to 5 years back) who we were targeting with the BPA and the non-BPA group (defined as eGFR 30–59 ml/min with ACR <300 mg/g up to 5 years back) who were not targeted by the BPA. Patients with CKD who were missing any albuminuria measurement within the past 5 years (initially 3%, which decreased to 0.9% by the end of the study) were included in the non-BPA group. We also examined prescribing trends in the BPA group, split by nephrology comanagement, and in patients not comanaged with nephrology, split by BPA versus non-BPA group. Break points were assessed using the Davies test, and slopes were calculated before and after the break points and assessed for statistical significance (defined as *P* < 0.05). We compared prescribing rates in patients not comanaged with nephrology, split by BPA versus non-BPA group, using multiple linear regression analysis adjusted for race, sex, age, CHF, and ASCVD. CHF and ASCVD were included as these were additional criteria for SGLT2i use separate from CKD in our formulary. Analysis was performed using R version 4.3.3 (The R Foundation, Free Software Foundation, Boston, MA).

## Results

### Baseline Characteristics

The number of patients in the diabetes registry at each point remained steady at around 40,000 throughout the study (Table 1 and Supplemental Table 2). Demographics were similar at each time point: average age was 61, 95% had DM2, 63%–65% had hypertension, 52%–53% were male, 71%–74% self-reported as White, 12%–13% had CKD, 4%–6% had CHF, and 15%–16% had ASCVD (Table 1 and Supplemental Table 2). Less than 0.01% of patients were missing sex or race. At baseline, 43% had ACR measured within 1 year, of which 63% had A1 albuminuria, and 79% had Scr checked within 1 year (Table 2). Patients with CKD in the BPA group were younger, and less likely to have hypertension, CHF, and ASCVD compared to the non-BPA group (Supplemental Table 10).

**Table 1:** Baseline characteristics in diabetes registry at beginning of study, before, and 1 and 2 years after implementation of clinical decision support tools

Characteristic	June 2021	May 2022	May 2023	May 2024
No.	39,952	40,376	40,844	42,076
Age (median)	61	61	61	62
DM2 (%)	95	95	95	95
**Race (%)**				
Asian	7	7	8	8
Black	4	5	5	5
Hispanic	9	10	10	10
Other	6	6	6	5
White	74	72	71	71
Sex (male, %)	52	53	52	52
Hypertension (%)	63	64	64	65
CKD (%)	12	13	12	12
CHF (%)	5	5	5	6
ASCVD (%)	16	15	15	16
eGFR (ml/min, median)	87	88	92	91
**ACR stage (%)^a^**				
A1	63	63	68	68
A2	20	20	21	22
A3	5	5	5	5
Unknown	12	12	7	5
ACEi/ARB use (%)	64	64	64	65
SGLT2i use (%)	2	3	6	10
Nephrology comanagement (%)	4	4	4	4

Patients younger than 18 years, older than 85 years, ESKD, on hospice or palliative care, and less than 1 year enrollment were excluded. ACEi, angiotensin-converting enzyme inhibitor; ACR, albumin-creatinine ratio; ARB, angiotensin receptor blocker; ASCVD, atherosclerotic cardiovascular disease; CHF, congestive heart failure; DM2, type 2 diabetes; SGLT2i, sodium-glucose cotransporter 2 inhibitor.

a Most recent albumin-creatinine ratio with look back up to 5 years from index date.

**Table 2:** Outcomes for eGFR testing within 1 year, albumin-creatinine ratio testing within 1 year, eGFR/albumin-creatinine ratio testing within 1 year, angiotensin-converting enzyme inhibitor/angiotensin receptor blocker use within 1 year in patients with angiotensin-converting enzyme inhibitor/angiotensin receptor blocker indication (defined as albumin-creatinine ratio ≥30 mg/g), sodium-glucose cotransporter 2 inhibitor use within 1 year in patients with sodium-glucose cotransporter 2 inhibitor indication (defined as type 2 diabetes with eGFR 30–59 ml/min or eGFR ≥30 ml/min and albumin-creatinine ratio ≥300 mg/g), and nephrology comanagement meeting referral criteria (eGFR <30 ml/min or albumin-creatinine ratio ≥1200 mg/g) at baseline, before, and 1 and 2 years after implementation of clinical decision support tools

Quality Metrics	June 2021	May 2022	May 2023	May 2024	*P* Value
eGFR (%)	31,500/39,952 (79)	30,761/40,376 (76)	34,480/40,844 (84)	35,951/42,076 (85)	<0.001
ACR (%)	17,237/39,952 (43)	16,558/40,376 (41)	30,692/40,844 (75)	31,830/42,076 (76)	<0.001
eGFR/ACR (%)	15,043/39,952 (38)	14,227/40,376 (35)	29,172/40,844 (71)	30,428/42,076 (72)	<0.001
ACEi/ARB use (%)	7173/9741 (74)	7220/9775 (74)	7879/10,514 (75)	8588/11,320 (76)	<0.001
SGLT2i use (%)	239/3868 (6)	497/4457 (11)	862/4173 (21)	1381/4271 (32)	<0.001
Nephrology comanagement (%)	453/842 (54)	444/845 (53)	419/809 (52)	433/818 (53)	0.87

ACEi, angiotensin-converting enzyme inhibitor; ACR, albumin-creatinine ratio; ARB, angiotensin receptor blocker; SGLT2i, sodium-glucose cotransporter 2 inhibitor.

### Outcomes

**Figure 1:**
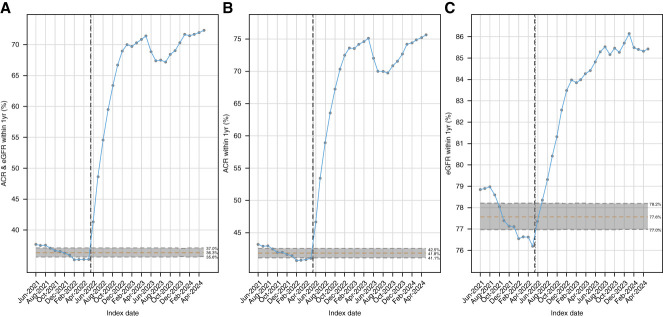
**Cumulative percentage of patients with diabetes with ACR, eGFR, or both within 1 year.** (A) P-chart of patients with diabetes who had both an ACR and eGFR measured within 1 year from index date. (B) P-chart of patients with diabetes who had an ACR measured within 1 year from index date. (C) P-chart of patients with diabetes who had an eGFR measured within 1 year from index date. Implementation of auto-lab ordering, printed reminders to providers, and automated patient reminders occurred in May 2022 (vertical line) with the red center horizontal line representing the preintervention mean and the upper and lower solid dashed lines representing three SDs from the mean. ACR, albumin-creatinine ratio.

One year after implementation of CDS tools, we were able to meet our target for ACR and Scr testing performed within 1 year and maintain this through end of follow-up (Figure 1A and Supplemental Table 3). Notably, ACR testing increased more than Scr testing (41%–76% versus 76%–85% by 1 year, respectively, Figure 1, B and C, and Table 2). Analysis of p-charts for ACR and Scr testing, as well as individual tests, showed a significant shift and trend following implementation of CDS tools, indicating a special cause variation.

**Figure 2:**
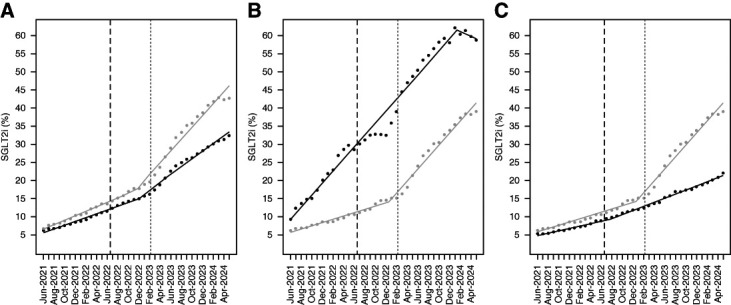
**Cumulative percentage of patients with DM2 prescribed SGLT2 inhibitor within 1 year.** (A) The percent of patients prescribed a SGLT2i within 1 year from index date in patients with CKD (eGFR 30–59 ml/min with any ACR or eGFR ≥60 ml/min with ACR ≥300 mg/g, black line) and patients with CKD in the BPA group (eGFR ≥30 ml/min and ACR ≥300 mg/g, gray line). (B) The percent of patients with CKD in the BPA group prescribed a SGLT2i within 1 year from index date split by comanagement with nephrology (black line) versus no comanagement with nephrology (gray line). (C) The percent of patients with CKD not comanaged with nephrology prescribed a SGLT2i within 1 year from index date split by the BPA group (gray line) versus the non-BPA group (CKD with ACR <300 and eGFR between 30 and 59 ml/min, black line). Black vertical line shows when the SGLT2i BPA was rolled out and gray vertical line when it was expanded to patients with ACR ≥300 mg/g and eGFR ≥60 ml/min. BPA, best practice alert; DM2, type 2 diabetes; SGLT2i, sodium-glucose cotransporter 2 inhibitor.

By end of follow up, SGLT2i use increased from 2% to 10% in patients with diabetes, from 6% to 32% in patients with diabetes and CKD, and from 7% to 43% in the BPA group (Table 1 and Supplemental Tables 6 and 7). The slope of SGLT2i prescribing in the BPA group increased from 0.6% per month to 1.7% per month 6 months after rollout of the CDS tools (Figure 2A and Table 3). When we compared the slope for SGLT2i use by nephrology comanagement, the slope remained mostly unchanged in the patients comanaged with nephrology (1.7% per month), while in the patients not comanaged with nephrology, the slope increased and matched the nephrology comanaged group after the breakpoint (0.5% per month to 1.7% per month, Figure 2B and Table 3). Among patients with CKD not comanaged with nephrology, we saw a significant increase in the slope for SGLT2i prescribing in both the BPA and non-BPA group after the breakpoint. However, the increase was larger in the BPA group compared with the non-BPA group (1.7% versus 0.6%, Figure 2C and Table 3). By the end of follow-up, the BPA group had a 7% increase in SGLT2i use compared with the non-BPA group after adjusting for age, sex, race, CHF, and ASCVD (*P* < 0.001, Table 4 and Supplemental Table 8).

**Table 3:** Slope for sodium-glucose cotransporter 2 inhibitor prescribing before and after the breakpoint in patients with type 2 diabetes and CKD

Group	Slope Before Breakpoint (% Change/Month with 95% CI)	Breakpoint	Slope after Breakpoint (% Change/Month with 95% CI)	*P* Value
CKD	0.5 (0.4 to 0.6)	November 20, 2022	1.1 (1.0 to 1.2)	<0.001
BPA CKD	0.6 (0.5 to 0.8)	November 22, 2022	1.7 (1.6 to 1.8)	<0.001
BPA CKD comanaged	1.7 (1.6 to 1.8)	January 16, 2024	−0.7 (−2.9 to 1.6)	0.008
**CKD non–comanaged**				
BPA	0.5 (0.4 to 0.6)	December 19, 2022	1.7 (1.6 to 1.8)	<0.001
Non-BPA	0.3 (0.3 to 0.4)	August 22, 2022	0.6 (0.5 to 0.6)	<0.001

The best practice alert CKD group consisted of patients with albumin-creatinine ratio ≥300 mg/g and eGFR ≥30 ml/min. The best practice alert CKD comanaged group consisted of the best practice alert CKD group followed by nephrology. The CKD non–co-managed group consisted of patients with CKD not comanaged by nephrology split by eGFR ≥30 ml/min with albumin-creatinine ratio ≥300 mg/g (best practice alert) versus eGFR 30–59 ml/min and albumin-creatinine ratio <300 mg/g (non–best practice alert). BPA, best practice alert; CI, confidence interval.

**Table 4:** Change in sodium-glucose cotransporter 2 inhibitor prescribing among patients not comanaged with nephrology in the best practice alert group (albumin-creatinine ratio ≥300 mg/g and eGFR ≥30 ml/min) versus the non–best practice alert group (eGFR 30–59 ml/min with albumin-creatinine ratio <300 mg/g) by end of follow-up

Group	Prior to SGLT2i BPA	After SGLT2i BPA	Difference	Adjusted Difference (95% CI)
BPA	10.7% (158/1483)	39% (636/1632)	28.3%	—
Non-BPA	8.9% (221/2477)	22% (459/2085)	13.1%	7% (6.2 to 7.8)

Sodium-glucose cotransporter 2 inhibitor best practice alert was rolled out on June 22, 2022. Difference adjusted for pre/post–clinical decision support rollout, age, sex, race, atherosclerotic cardiovascular disease, and congestive heart failure. BPA, best practice alert; CI, confidence interval; SGLT2i, sodium-glucose cotransporter 2 inhibitor.

Within the BPA group, initially only 21% of patients were triggering the BPA, while 65% were not. A review in October 2022 revealed that a significant number of patients with eGFR ≥60 ml/min and ACR ≥300 mg/g were not firing the BPA due to lack of documented CKD diagnosis (Supplemental Figure 2). To address this, we modified the BPA by removing the requirement for CKD diagnosis which went into effect in February 2023. This resulted in a substantial increase in the percent of patients triggering the BPA, with 48% activating the alert and 31% remaining untriggered. Furthermore, the proportion of patients who did not fire the BPA due to missing ACR or eGFR within 1 year decreased from 33% to 15% by end of follow-up.

**Figure 3:**
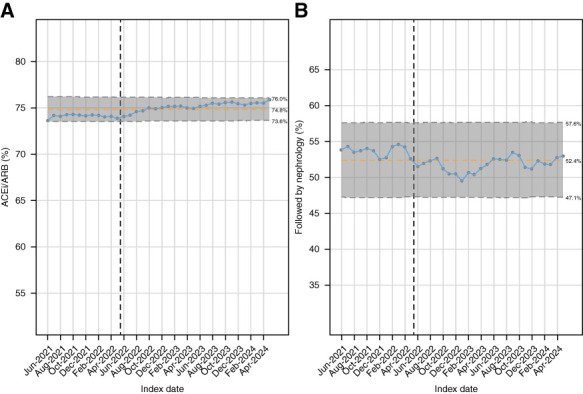
**Cumulative percentage of patients prescribed ACEi/ARB or seen by nephrology within 1 year.** (A) P-chart of patients with diabetes with last ACR ≥30 mg/g who had an ACEi or ARB ordered within 1 year from index date. (B) P-chart of patients with diabetes with CKD and last eGFR <30 ml/min or last ACR ≥1200 mg/g seen by nephrology within 1 year. Implementation of auto-lab ordering, printed reminders to providers, and automated patient reminders occurred in May 2022 (vertical line) with the red center horizontal line representing the mean and the upper and lower dashed horizontal lines representing three SDs from the mean. ACEi, angiotensin-converting enzyme inhibitor; ARB, angiotensin receptor blocker.

The overall rate of ACEi/ARB use increased slightly from 64% to 65% (Table 1). The percentage of patients with diabetes who met criteria for ACEi/ARB use increased from 24% to 27% by end of the study, and we saw a small but statistically significant increase in ACEi/ARB use for in patients with renal indications (74%–76%, *P* < 0.001, Figure 3A, Table 2, and Supplemental Table 4). Patients not prescribed an ACEi/ARB were more likely to be younger, female, and less likely to have hypertension, ASCVD, and CKD (Supplemental Table 9). Comanagement of patients meeting nephrology referral criteria was constant at 53% (Figure 3B, Table 2, and Supplemental Table 5). In patients meeting criteria for nephrology referral, worsening CKD stages was associated with higher rates of nephrology comanagement (Supplemental Figure 3). For balancing measures, we saw an increase in patients with controlled BP and retinal screening but no change in diabetes control after rollout of the CDS tools (Supplemental Figure 4).

## Discussion

Our QI project successfully increased ACR and Scr testing within 1 year in patients with diabetes using CDS tools. We showed that the addition of patient facing reminders with laboratory automation increased screening for CKD. A key strength of our study was the successful implementation of CDS tools in a large population using automation of laboratory results without reliance on staff to enter orders. In addition, with increased testing, we did not see any negative effect on other diabetes quality metrics that we were able to track. Our results mirror another large health care system that used automated laboratory ordering to screen 78% of patients with diabetes for CKD.^28^ Other QI projects have successfully increased CKD screening in patients with diabetes using CDS tools but at a smaller scale and using different approaches. Kam *et al.* implemented a QI project in which recommendations for ACR testing were included in the EHR dashboard and ordered by staff for the provider to sign, which increased ACR testing by 12.7%.^29^ Anabtawi *et al.* increased ACR testing from 56% to 84% by implementing a BPA about diabetes care guidelines.^30^

The effect of CDS tools on other CKD quality metrics varied. Notably, we saw a steeper increase in SGLT2i prescribing rates among patients in the BPA group, suggesting the potential of this intervention to improve CKD outcomes. Although SGLT2i utilization also increased among patients with CKD who were in the non-BPA group, likely due to growing provider familiarity and comfort with prescribing SGLT2i, the rate of increase was more pronounced in the BPA group. Importantly, this upward trend was similar regardless of whether patients were comanaged with nephrology. By contrast to ACR and Scr testing, the effect of CDS tools on SGLT2i use was not immediate. This delay can be attributed to several factors, including a potential lag in provider recognition of SGLT2i benefits in patients with early CKD who were initially excluded from the BPA and clinical inertia which has been shown to occur in diabetes management.^31–33^ Moreover, the BPA would only be seen at time of office visits, which occurred at varying intervals. Studies combining provider education with CDS tools or provider audits have successfully increased SGLT2i use. For example, Ghazi *et al.* observed a 7% increase in guideline-medical directed therapy among CHF patients using BPAs, including a modest increase in SGLT2i.^16^ Similarly, Pagidipati *et al.* successfully increased SGLT2i use in patients with ASCVD using a multicomponent intervention that included provider education and audits.^34^

Despite increased ACR and Scr testing, ACEi/ARB prescribing rates increased only marginally. The effect was likely limited as the recommendation for ACEi/ARB use had already been in place since 2009. Previous studies evaluating CDS tools to increase ACEi/ARB use have shown mixed results. Sequist *et al.* found no difference in ACEi/ARB use among patients with stage 3 CKD whose primary care physicians receive BPAs.^35^ Similarly, Sperl-hillen *et al.* and Peralta *et al.* reported no significant increase in ACEi/ARB initiation among patients with CKD despite the use of BPAs and printed recommendations.^20,36^ However, other studies incorporating “behavioral nudges” alongside CDS tools have shown more promising results. Tuot *et al.* observed a two-fold increase in ACEi/ARB use by providing quarterly feedback to providers in conjunction with BPAs.^17^ Vazquez *et al.* increased ACEi/ARB use in patients with CKD and diabetes through a multifaceted intervention combining BPAs linked to smart sets with practice facilitator support, including medication management and feedback.^37^ Jhamb *et al.* increased ACEi/ARB use in patients with high-risk CKD using BPAs with recommendations provided through chart review by a nephrologist and pharmacist.^19^ Samal *et al.* increased ACEi/ARB use and up-titration by incorporating precommitment emails alongside BPAs linked to smart set recommendations for patients with uncontrolled hypertension and CKD.^18^

We did not see an increase in referrals of high-risk patients with CKD to nephrology despite an increase in ACR and Scr testing. This lack of improvement may be attributed to reliance solely on clinician education. We found lower rates of nephrology visits in earlier CKD stages consistent with other studies showing that many primary care providers recognize eGFR as a marker of high-risk CKD but may not adequately consider albuminuria.^38,39^ Previous studies evaluating BPAs to increase nephrology referral have yielded mixed results. Although Sequist *et al.* successfully increased referrals using a BPA for high-risk CKD, other studies did not observe a significant effect on referral rates despite the use of BPAs.^20,35,36,40^

Our study had several limitations. We did not assess the individual effect of each intervention on CKD screening and could not track how frequently providers interacted with each intervention, limiting our understanding of their utilization. As these efforts are resource intensive, it would be of interest in future studies to assess which intervention would likely be used by clinicians and affect CKD screening the most. We did not access the effect of these interventions on clinician workload, which may have included increased review of laboratory results, patient communication, redundant testing in patients who had a urine protein checked, and inappropriate recommendations in patients with transient albuminuria. Furthermore, our study design limits our ability to establish causality between the interventions and SGLT2i prescribing, and several factors may also have confounded our analysis. The effect of the BPA may be underestimated as the BPA did not fire in a significant proportion in the BPA targeted group. In addition, some patients treated with SGLT2i in the BPA group may have been subsequently reclassified into the non-BPA group due to regression of albuminuria. Furthermore, a small number of patients in the non-BPA group were targeted by a separate BPA in a concurrent project targeting patients with heart failure with reduced ejection fraction. Finally, although our analysis tried to adjust for other indications for SGLT2i use, we did not account for other potential confounders such as patient income and diabetes control.

The generalizability of our QI project to other health care systems may be limited. Our QI project required resources to build BPAs, disease registries, and automate laboratory ordering and patient outreach which may not be available in many health care systems. In addition, as KPNW is a closed integrated system which allows for easier implementation of CDS tools, enacting these tools would be more challenging in nonintegrated health care systems where it would be more difficult to track laboratory results and medications. In addition, the predominantly White study population limits the generalizability of the findings to more diverse populations, as previous studies have shown lower SGLT2i use in Black and Hispanic patients.^41^

This study demonstrates that implementation of CDS tools can effectively improve CKD screening in patients with diabetes and sustain these improvements over time. Although these tools have the potential to be adapted and implemented in other integrated health care systems, further research is needed to determine their effectiveness in less integrated health care systems. More critically, given the emergence of novel therapies for CKD, future cluster randomized controlled trials are imperative to evaluate whether CDS tools can reduce CKD progression and MACE.

## Supplementary Material

## Data Availability

Partial restrictions to the data and/or materials apply. The data for this study is available upon request, but requests need to be reviewed and approved by Kaiser Permanente and Centers for Health Research. Requests can be sent to the corresponding author.

## Supplemental Material

Supplemental Table 1. ICD-10 codes for CHF and ASCVD.

Supplemental Table 2. Baseline demographics by index date in diabetes registry.

Supplemental Table 3. Cumulative percentage of patients with diabetes that had urine ACR ratio, eGFR, or both within 1 year.

Supplemental Table 4. Cumulative percentage of patients with diabetes prescribed an ACEi or ARB with renal indication.

Supplemental Table 5. Cumulative percentage of patients with diabetes with most recent ACR ≥1200 mg/g or eGFR <30 ml/min with CKD seen by nephrology.

Supplemental Table 6. Cumulative percentage of patients with DM2 with CKD who were prescribed a SGLT2i if indicated.

Supplemental Table 7. Cumulative percentage of patients with DM2 targeted by BPA prescribed SGLT2i.

Supplemental Table 8. Multiple linear regression model for outcome of SGLT2i use divided by BPA pre-CDS and post-CDS rollout.

Supplemental Table 9. Patient characteristics with renal indication for ACEi/ARB, stratified by ACEi/ARB prescription status.

Supplemental Table 10. Characteristics in patients with CKD and DM2, stratified by whether they were targeted by BPA for SGLT2i.

Supplemental Figure 1. Timeline of QI interventions.

Supplemental Figure 2. Bar plot of patients with CKD and DM2 in the BPA group who were excluded from the BPA firing due to missing ACR/eGFR or contraindications.

Supplemental Figure 3. Bar plot of patients with CKD with indication for nephrology comanagement seen by nephrology within 1 year.

Supplemental Figure 4. P-chart of patients with diabetes with controlled diabetes, controlled BP, and retinal screening done within 1 year.
