# Supplementary material for: Improving CKD Screening and Care in Diabetes Using Clinical Decision Support in a Large Health Care System
Source: Kidney360. 2025 Apr 24;6(9):1501–9. doi: 10.34067/KID.0000000829 (PMC12483030; doi:10.34067/KID.0000000829)
Supplement: Supplementary file 1 [file kidney360-6-01501-s001.pdf]

ASN American Society of Nephrology JASN Journal of the American Society of Nephrology KIDNEY360 Accessing Our World From Every Angle CJASN Clinical Journal of the American Society of Nephrology

ASN Journal Disclosure Form

As per ASN journal policy, I have disclosed any financial relationships or commitments I have held in the past 36 months as included below. I have listed my Current Employer below to indicate there is a relationship requiring disclosure. If no relationship exists, my Current Employer is not listed.

E. Albright reports the following:
Employer: Kaiser Permanenete

I understand that the information above will be published within the journal article, if accepted, and that failure to comply and/or to accurately and completely report the potential financial conflicts of interest could lead to the following: 1) Prior to publication, article rejection, or 2) Post-publication, sanctions ranging from, but not limited to, issuing a correction, reporting the inaccurate information to the authors' institution, banning authors from submitting work to ASN journals for varying lengths of time, and/or retraction of the published work.

Name: Eric S. Albright
Manuscript ID: K360-2024-000730R1
Manuscript Title: Improving chronic kidney disease screening and care in diabetes using clinical decision support in a large healthcare system
Date of Completion: January 23, 2025
Disclosure Updated Date: January 23, 2025

ASN American Society of Nephrology | JASN Journal of the American Society of Nephrology | KIDNEY360 Accessing Our World From Every Angle | CJASN Clinical Journal of the American Society of Nephrology

ASN Journal Disclosure Form

As per ASN journal policy, I have disclosed any financial relationships or commitments I have held in the past 36 months as included below. I have listed my Current Employer below to indicate there is a relationship requiring disclosure. If no relationship exists, my Current Employer is not listed.

F. Chen reports the following:
Employer: Kaiser Permanente (aka Northwest Permanente)

I understand that the information above will be published within the journal article, if accepted, and that failure to comply and/or to accurately and completely report the potential financial conflicts of interest could lead to the following: 1) Prior to publication, article rejection, or 2) Post-publication, sanctions ranging from, but not limited to, issuing a correction, reporting the inaccurate information to the authors' institution, banning authors from submitting work to ASN journals for varying lengths of time, and/or retraction of the published work.

Name: Frank Chen
Manuscript ID: K360-2024-000730R1
Manuscript Title: Improving chronic kidney disease screening and care in diabetes using clinical decision support in a large healthcare system
Date of Completion: March 3, 2025
Disclosure Updated Date: March 3, 2025

ASN American Society of Nephrology
JASN Journal of the American Society of Nephrology
KIDNEY360 Accessing Our World From Every Angle
CJASN Clinical Journal of the American Society of Nephrology

ASN Journal Disclosure Form

As per ASN journal policy, I have disclosed any financial relationships or commitments I have held in the past 36 months as included below. I have listed my Current Employer below to indicate there is a relationship requiring disclosure. If no relationship exists, my Current Employer is not listed.

S. Flerchinger has nothing to disclose.

I understand that the information above will be published within the journal article, if accepted, and that failure to comply and/or to accurately and completely report the potential financial conflicts of interest could lead to the following: 1) Prior to publication, article rejection, or 2) Post-publication, sanctions ranging from, but not limited to, issuing a correction, reporting the inaccurate information to the authors' institution, banning authors from submitting work to ASN journals for varying lengths of time, and/or retraction of the published work.

Name: Shaun Flerchinger
Manuscript ID: K360-2024-000730R1
Manuscript Title: Improving chronic kidney disease screening and care in diabetes using clinical decision support in a large healthcare system
Date of Completion: January 23, 2025
Disclosure Updated Date: January 23, 2025

ASN American Society of Nephrology | JASN Journal of the American Society of Nephrology | KIDNEY360 Accessing Our World From Every Angle | CJASN Clinical Journal of the American Society of Nephrology

ASN Journal Disclosure Form

As per ASN journal policy, I have disclosed any financial relationships or commitments I have held in the past 36 months as included below. I have listed my Current Employer below to indicate there is a relationship requiring disclosure. If no relationship exists, my Current Employer is not listed.

K. Glassberg reports the following:
Employer: Northwest Permanente; and Ownership Interest: Eli Lilly (inherited and sold quickly); Intel Corp (my husband's employer and part of his remuneration).

I understand that the information above will be published within the journal article, if accepted, and that failure to comply and/or to accurately and completely report the potential financial conflicts of interest could lead to the following: 1) Prior to publication, article rejection, or 2) Post-publication, sanctions ranging from, but not limited to, issuing a correction, reporting the inaccurate information to the authors' institution, banning authors from submitting work to ASN journals for varying lengths of time, and/or retraction of the published work.

Name: Kathryn Glassberg
Manuscript ID: K360-2024-000730R1
Manuscript Title: Improving chronic kidney disease screening and care in diabetes using clinical decision support in a large healthcare system
Date of Completion: April 10, 2025
Disclosure Updated Date: April 10, 2025

ASN Journal Disclosure Form

As per ASN journal policy, I have disclosed any financial relationships or commitments I have held in the past 36 months as included below. I have listed my Current Employer below to indicate there is a relationship requiring disclosure. If no relationship exists, my Current Employer is not listed.

M. Tandy reports the following:
Employer: Northwest Permanente

I understand that the information above will be published within the journal article, if accepted, and that failure to comply and/or to accurately and completely report the potential financial conflicts of interest could lead to the following: 1) Prior to publication, article rejection, or 2) Post-publication, sanctions ranging from, but not limited to, issuing a correction, reporting the inaccurate information to the authors' institution, banning authors from submitting work to ASN journals for varying lengths of time, and/or retraction of the published work.

Name: Michalah Tandy
Manuscript ID: K360-2024-000730R1
Manuscript Title: Improving chronic kidney disease screening and care in diabetes using clinical decision support in a large healthcare system
Date of Completion: February 3, 2025
Disclosure Updated Date: February 3, 2025

ASN American Society of Nephrology | JASN Journal of the American Society of Nephrology | KIDNEY360 Accessing Our World From Every Angle | CJASN Clinical Journal of the American Society of Nephrology

ASN Journal Disclosure Form

As per ASN journal policy, I have disclosed any financial relationships or commitments I have held in the past 36 months as included below. I have listed my Current Employer below to indicate there is a relationship requiring disclosure. If no relationship exists, my Current Employer is not listed.

L. Nakashimada reports the following:
Employer: Kaiser Foundation Health Plan of the Northwest

I understand that the information above will be published within the journal article, if accepted, and that failure to comply and/or to accurately and completely report the potential financial conflicts of interest could lead to the following: 1) Prior to publication, article rejection, or 2) Post-publication, sanctions ranging from, but not limited to, issuing a correction, reporting the inaccurate information to the authors' institution, banning authors from submitting work to ASN journals for varying lengths of time, and/or retraction of the published work.

Name: Lisa J. Nakashimada
Manuscript ID: K360-2024-000730R1
Manuscript Title: Improving chronic kidney disease screening and care in diabetes using clinical decision support in a large healthcare system
Date of Completion: January 24, 2025
Disclosure Updated Date: January 24, 2025

ASN American Society of Nephrology
JASN Journal of the American Society of Nephrology
KIDNEY360 Accessing Our World From Every Angle
CJASN Clinical Journal of the American Society of Nephrology

ASN Journal Disclosure Form

As per ASN journal policy, I have disclosed any financial relationships or commitments I have held in the past 36 months as included below. I have listed my Current Employer below to indicate there is a relationship requiring disclosure. If no relationship exists, my Current Employer is not listed.

K. Park reports the following:
Employer: Kaiser Permanente

I understand that the information above will be published within the journal article, if accepted, and that failure to comply and/or to accurately and completely report the potential financial conflicts of interest could lead to the following: 1) Prior to publication, article rejection, or 2) Post-publication, sanctions ranging from, but not limited to, issuing a correction, reporting the inaccurate information to the authors' institution, banning authors from submitting work to ASN journals for varying lengths of time, and/or retraction of the published work.

Name: Ken J. Park
Manuscript ID: K360-2024-000730
Manuscript Title: A quality improvement project to increase chronic kidney disease screening and SGLT2-inhibitor use in patients with diabetes
Date of Completion: January 20, 2025
Disclosure Updated Date: January 20, 2025
