# Supplementary material for: Improving CKD Screening and Care in Diabetes Using Clinical Decision Support in a Large Health Care System
Source: Kidney360. 2025 Apr 24;6(9):1501–9. doi: 10.34067/KID.0000000829 (PMC12483030; doi:10.34067/KID.0000000829)

Supplemental Material

Table of contents

**Title**	**Page**
Supplemental Table 1. ICD-10 codes for CHF and ASCVD	2
Supplemental Table 2. Baseline demographics by index date in diabetes registry.	3
Supplemental Table 3. Cumulative percentage of patients with diabetes that had urine albumin creatinine ratio, eGFR, or both within 1 yr	5
Supplemental Table 4. Cumulative percentage of patients with diabetes prescribed an ACEi or ARB with renal indication	7
Supplemental Table 5. Cumulative percentage of patients with diabetes with most recent ACR ≥ 1200 mg/gm or eGFR < 30 ml/min with CKD seen by nephrology	8
Supplemental Table 6. Cumulative percentage of patients with type 2 diabetes with CKD indication for SGLT2i prescribed SGLT2-inhibitor	9
Supplemental Table 7. Cumulative percentage of patients with type 2 diabetes targeted by BPA prescribed SGLT2-inhibitor	10
Supplemental Table 8. Multiple linear regression model for outcome of SGLT2-inhibitor use divided by BPA pre and post CDS rollout	11
Supplemental Table 9. Characteristics in patients with renal indication for ACEi or ARB divided by whether patient prescribed ACEi or ARB	12
Supplemental Table 10. Characteristics in patients with CKD and DM2 divided by whether patients were targeted by BPA for SGLT2-inhibitor	13
Supplemental Figure 1. Timeline of quality improvement interventions	14
Supplemental Figure 2: Bar plot of patients with CKD and DM2 targeted by the BPA who were excluded from the BPA firing due to missing ACR/eGFR or contraindications.	15
Supplemental Figure 3: Bar plot of patients with CKD with indication for nephrology co-management seen by nephrology within 1 year.	16
Supplemental Figure 4: P-chart of patients with diabetes with controlled diabetes, controlled blood pressure, and retinal screening done within 1 year.	17

Supplemental Table 1. ICD-10 codes for CHF and ASCVD

CHF	I09.81, I11.0, I13.0, I13.2, I25.5, I27.29, I42.0, I42.5, I42.9, I50.1, I50.20, I50.21, I50.22, I50.23, I50.30, I50.31, I50.32, I50.33, I50.40, I50.41, I50.42, I50.43, I50.810, I50.811, I50.812, I50.813, I50.814, I50.82, I50.83, I50.84, I50.89, I50.9, I51.9, R57.0, Z91.89
ASCVD	E10.59, E11.59, G45.xx, I15.0, I20.xx, I12.xxx, I22.xx, I23.7, I24.xx, I25.10, I125.11x, 125.2, I25.5, I50.84, I25.6, I25.7xx, I25.8xx, I25.9, I63.xxx, I65.xx, I66.xx, I67.2, I67.81, I67.82, I67.841, I70.1, I70.2xx, I70.92, I73.9, I75.xxx, I77.1, I77.9, I79.8, V45.09, V45.88, Z95.1, Z95.5, Z98.61, Z98.62, Z98.89

Supplemental Table 2. Baseline demographics by index date in diabetes registry. Patients less than 18, older than 85, end stage renal disease, on hospice or palliative care, and less than 1 year enrollment were excluded.

Index date	6/5/2021	7/5/2021	8/5/2021	9/5/2021	10/5/2021	11/5/2021	12/5/2021	1/5/2022	2/5/2022	3/5/2022	4/5/2022	5/5/2022
Number	39,952	39,923	39,981	39,975	39,947	40,764	40,702	40,133	40,121	40,153	40,261	40,376
Age (median)	61	61	61	61	61	61	61	61	61	61	61	61
Type 2 diabetes (%)	95	95	95	95	95	95	95	95	95	95	95	95
Race (%)	Asian 7<br>Black 4<br>Hispanic 9<br>White 74<br>Other 6	Asian 7<br>Black 4<br>Hispanic 9<br>White 74<br>Other 6	Asian 7<br>Black 4<br>Hispanic 9<br>White 73<br>Other 7	Asian 7<br>Black 4<br>Hispanic 10<br>White 73<br>Other 6	Asian 7<br>Black 4<br>Hispanic 10<br>White 73<br>Other 6	Asian 7<br>Black 4<br>Hispanic 10<br>White 73<br>Other 6	Asian 7<br>Black 4<br>Hispanic 10<br>White 73<br>Other 6	Asian 7<br>Black 5<br>Hispanic 10<br>White 73<br>Other 5	Asian 7<br>Black 5<br>Hispanic 10<br>White 73<br>Other 5	Asian 7<br>Black 5<br>Hispanic 10<br>White 72<br>Other 6	Asian 7<br>Black 5<br>Hispanic 10<br>White 72<br>Other 6	Asian 7<br>Black 5<br>Hispanic 10<br>White 72<br>Other 6
Gender (M, %)	52	52	52	52	52	52	52	52	52	53	53	53
Htn (%)	63	63	63	63	63	63	63	64	64	64	64	64
CKD (%)	12	12	12	13	13	13	13	13	13	13	13	13
CHF (%)	5	5	5	5	5	5	5	5	5	5	5	5
ASCVD (%)	16	16	16	16	16	15	15	15	15	15	15	15

Index date	6/5/2022	7/5/2022	8/5/2022	9/5/2022	10/5/2022	11/5/2022	12/5/2022	1/5/2023	2/5/2023	3/5/2023	4/5/2023	5/5/2023
Number	40,500	40,553	40,673	40,630	40,586	41,187	41,130	40,538	40,553	40,632	40,731	40,844
Age (median)	61	61	61	61	61	61	61	61	61	61	61	61
Type 2 DM (%)	95	95	95	95	95	95	95	95	95	95	95	95
Race (%)	Asian 7<br>Black 5<br>Hispanic 10<br>White 72<br>Other 6	Asian 7<br>Black 5<br>Hispanic 10<br>White 72<br>Other 6	Asian 7<br>Black 5<br>Hispanic 10<br>White 72<br>Other 6	Asian 7<br>Black 5<br>Hispanic 10<br>White 72<br>Other 6	Asian 8<br>Black 5<br>Hispanic 10<br>White 72<br>Other 5	Asian 8<br>Black 5<br>Hispanic 10<br>White 72<br>Other 5	Asian 8<br>Black 5<br>Hispanic 10<br>White 71<br>Other 6	Asian 8<br>Black 5<br>Hispanic 10<br>White 71<br>Other 6	Asian 8<br>Black 5<br>Hispanic 10<br>White 71<br>Other 6	Asian 8<br>Black 5<br>Hispanic 10<br>White 71<br>Other 6	Asian 8<br>Black 5<br>Hispanic 10<br>White 71<br>Other 6	Asian 8<br>Black 5<br>Hispanic 10<br>White 71<br>Other 6
Gender (M, %)	53	53	53	53	53	53	52	52	52	52	52	52
Htn (%)	64	64	64	64	64	64	64	64	64	64	64	64
CKD (%)	13	13	13	13	13	13	13	13	12	12	12	12
CHF (%)	5	5	5	4	5	4	4	5	5	5	5	4

ASCVD (%)	15	15	15	15	15	15	15	15	15	15	15	15

Index date	6/5/2023	7/5/2023	8/5/2023	9/5/2023	10/5/2023	11/5/2023	12/5/2023	1/5/2024	2/5/2024	3/5/2024	4/5/2024	5/5/2024
Number	40,938	40,978	40,986	40,014	40,937	41,456	42,721	41,479	41,601	41,660	41,951	42,076
Age (median)	61	61	61	61	61	61	62	62	62	62	62	62
Type 2 DM (%)	95	95	95	95	95	95	95	95	95	95	95	95
Race (%)	Asian 8<br>Black 5<br>Hispanic 10<br>White 71<br>Other 6	Asian 8<br>Black 5<br>Hispanic 10<br>White 71<br>Other 6	Asian 8<br>Black 5<br>Hispanic 10<br>White 71<br>Other 6	Asian 8<br>Black 5<br>Hispanic 10<br>White 71<br>Other 6	Asian 8<br>Black 5<br>Hispanic 10<br>White 71<br>Other 5	Asian 8<br>Black 5<br>Hispanic 11<br>White 71<br>Other 5	Asian 8<br>Black 5<br>Hispanic 10<br>White 71<br>Other 6	Asian 8<br>Black 5<br>Hispanic 10<br>White 71<br>Other 6	Asian 8<br>Black 5<br>Hispanic 10<br>White 71<br>Other 6	Asian 8<br>Black 5<br>Hispanic 10<br>White 71<br>Other 5	Asian 8<br>Black 5<br>Hispanic 10<br>White 71<br>Other 6	Asian 8<br>Black 5<br>Hispanic 10<br>White 71<br>Other 5
Gender (M, %)	52	52	52	52	52	52	52	52	52	52	52	52
Htn (%)	64	64	64	64	64	64	65	65	65	65	65	65
CKD (%)	12	12	12	12	12	12	12	12	12	12	12	12
CHF (%)	5	5	5	5	5	5	5	5	5	5	6	6
ASCVD (%)	15	15	15	15	15	15	15	16	16	16	16	16

Supplemental Table 3. Cumulative percentage of patients with diabetes that had urine albumin creatinine ratio (ACR), eGFR, or both checked within 1 year of index date. Auto-lab ordering, printed reminders to providers, and automated patient reminders were implemented in May 2022.

Index date	6/5/2021	7/5/2021	8/5/2021	9/5/2021	10/5/2021	11/5/2021	12/5/2021	1/5/2022	2/5/2022	3/5/2022	4/5/2022	5/5/2022
Number	39,952	39,923	39,981	39,975	39,947	40,764	40,702	40,133	40,121	40,153	40,261	40,376
eGFR (n)	31,500	31,496	31,573	31,416	31,179	31,547	31,390	30,942	30,712	30,766	30,842	30,761
eGFR (%)	79	79	79	79	78	78	77	77	77	77	77	76
ACR (n)	17,237	17,124	17,154	16,964	16,744	17,100	16,955	16,619	16,296	16,350	16,431	16,558
ACR (%)	43	43	43	42	42	42	42	41	41	41	41	41
ACR & eGFR (n)	15,043	14,969	15,002	14,828	14,629	14,887	14,758	14,444	14,107	14,150	14,192	14,227
ACR & eGFR (%)	38	38	38	37	37	37	36	36	35	35	35	35

Index date	6/5/2022	7/5/2022	8/5/2022	9/5/2022	10/5/2022	11/5/2022	12/5/2022	1/5/2023	2/5/2023	3/5/2023	4/5/2023	5/5/2023
Number	40,500	40,553	40,673	40,630	40,586	41,187	41,130	40,538	40,553	40,632	40,731	40,844
eGFR (n)	31,327	31,768	32,257	32,671	33,006	34,008	34,339	34,042	34,001	34,122	34,321	34,480
eGFR (%)	77	78	79	80	81	83	84	84	84	84	84	84
ACR (n)	18,888	21,668	23,962	25,805	27,287	28,962	29,823	29,839	29,829	30,147	30,380	30,692
ACR (%)	47	53	59	64	67	70	73	74	74	74	75	75
ACR & eGFR (n)	16,372	19,708	22,189	24,174	25,728	27,470	28,356	28,368	28,251	28,566	28,858	29,172
ACR & eGFR (%)	41	49	55	60	63	67	69	70	70	70	71	71

Index date	6/5/2023	7/5/2023	8/5/2023	9/5/2023	10/5/2023	11/5/2023	12/5/2023	1/5/2024	2/5/2024	3/5/2024	4/5/2024	5/5/2024
Number	40,938	40,978	40,986	40,014	40,937	41,456	42,721	41,479	41,601	41,660	41,951	42,076
eGFR (n)	34,722	34,950	35,053	34,935	34,979	35,351	36,615	35,730	35,562	35,581	35,800	35,951

eGFR (%)	85	85	86	85	85	85	86	86	86	85	85	85
ACR (n)	29,478	28,702	28,688	28,600	29,015	29,656	31,044	30,772	30,952	31,168	31,571	31,830
ACR (%)	72	70	70	70	71	72	73	74	74	75	75	76
ACR & eGFR (n)	28,197	27,618	27,662	27,564	27,997	28,625	30,028	29,723	29,724	29,846	30,183	30,428
ACR & eGFR (%)	69	67	68	67	68	70	70	72	72	72	72	72

Supplemental Table 4. Cumulative percentage of patients with diabetes with most recent ACR > 30 mg/gm (up to 5 years lookback) prescribed an ACEi or ARB within 1 year of index date. Auto-lab ordering, printed reminders to providers, and automated patient reminders were implemented in May 2022.

Index date	6/5/2021	7/5/2021	8/5/2021	9/5/2021	10/5/2021	11/5/2021	12/5/2021	1/5/2022	2/5/2022	3/5/2022	4/5/2022	5/5/2022
Number	9,741	9,673	9,682	9,614	9,610	9,846	9,823	9,693	9,747	9,759	9,786	9,775
Percent of cohort (%)	24	24	24	24	24	24	24	24	24	24	24	24
Prescribed ACEi or ARB (n)	7,173	7,174	7,172	7,136	7,140	7,308	7,280	7,195	7,228	7,225	7,248	7,220
Prescribed ACEi or ARB (%)	74	74	74	74	74	74	74	74	74	74	74	74

Index date	6/5/2022	7/5/2022	8/5/2022	9/5/2022	10/5/2022	11/5/2022	12/5/2022	1/5/2023	2/5/2023	3/5/2023	4/5/2023	5/5/2023
Number	9,950	10,116	10,245	10,258	10,294	10,557	10,583	10,510	10,533	10,557	10,531	10,514
Percent of cohort (%)	25	25	25	25	25	26	26	26	26	26	26	26
Prescribed ACEi or ARB (n)	7,373	7,511	7,643	7,662	7,719	7,907	7,936	7,897	7,915	7,939	7,897	7,879
Prescribed ACEi or ARB (%)	74	74	75	75	75	75	75	75	75	75	75	75

Index date	6/5/2023	7/5/2023	8/5/2023	9/5/2023	10/5/2023	11/5/2023	12/5/2023	1/5/2024	2/5/2024	3/5/2024	4/5/2024	5/5/2024
Number	10,572	10,591	10,574	10,578	10,634	10,799	11,328	11,101	11,148	11,161	11,272	11,320
Percent of cohort (%)	26	26	26	26	26	26	27	27	27	27	27	27
Prescribed ACEi or ARB (n)	7,942	7,977	7,985	7,975	8,037	8,165	8,546	8,359	8,414	8,432	8,511	8,588
Prescribed ACEi or ARB (%)	75	75	76	75	76	76	75	75	76	76	76	76

Supplemental Table 5. Cumulative percentage of patients with diabetes with most recent ACR ≥ 1200 mg/gm or eGFR < 30 ml/min with CKD (up to 5 years lookback) seen by nephrology within 1 year. Auto-lab ordering, printed reminders to providers, and automated patient reminders were implemented in May 2022

Index date	6/5/2021	7/5/2021	8/5/2021	9/5/2021	10/5/2021	11/5/2021	12/5/2021	1/5/2022	2/5/2022	3/5/2022	4/5/2022	5/5/2022
Number	842	831	828	823	815	832	829	816	814	822	834	845
Percent of cohort (%)	2	2	2	2	2	2	2	2	2	2	2	2
Seen by nephrology (n)	453	451	443	442	440	447	435	430	442	449	452	444
Seen by nephrology (%)	54	54	54	54	54	54	52	53	54	55	54	53

Index date	6/5/2022	7/5/2022	8/5/2022	9/5/2022	10/5/2022	11/5/2022	12/5/2022	1/5/2023	2/5/2023	3/5/2023	4/5/2023	5/5/2023
Number	839	821	824	832	817	831	823	817	812	812	816	809
Percent of cohort (%)	2	2	2	2	2	2	2	2	2	2	2	2
Seen by nephrology (n)	432	426	431	438	418	419	415	404	411	409	418	419
Seen by nephrology (%)	51	52	52	53	51	50	50	49	51	50	51	52

Index date	6/5/2023	7/5/2023	8/5/2023	9/5/2023	10/5/2023	11/5/2023	12/5/2023	1/5/2024	2/5/2024	3/5/2024	4/5/2024	5/5/2024
Number	826	810	815	813	811	796	846	847	841	844	826	818
Percent of cohort (%)	2	2	2	2	2	2	2	2	2	2	2	2
Seen by nephrology (n)	434	425	427	435	430	409	433	443	436	437	436	433
Seen by nephrology (%)	53	52	52	54	53	51	51	52	52	52	53	53

Supplemental Table 6. Cumulative percentage of patients with type 2 diabetes with CKD indication for SGLT2i (last ACR > 300 mg/gm or CKD with last eGFR 30-59 ml/min up to 5 years lookback) prescribed SGLT2-inhibitor within 1 year of index date. BPA recommending SGLT2-inhibitor in patients with ACR > 300 mg/gm and eGFR ≥ 30 ml/min was implemented in June 2022 and expanded to include patients with eGFR ≥ 60 ml/min in February 2023.

Index date	6/5/2021	7/5/2021	8/5/2021	9/5/2021	10/5/2021	11/5/2021	12/5/2021	1/5/2022	2/5/2022	3/5/2022	4/5/2022	5/5/2022
Number	3,868	3,993	4,173	4,227	4,290	4,444	4,477	4,444	4,468	4,476	4,503	4,457
Percent of cohort (%)	10	10	10	11	11	11	11	11	11	11	11	11
Prescribed SGLT2i (n)	239	268	284	299	325	352	378	387	408	443	472	497
Prescribed SGLT2i (%)	6	7	7	7	8	8	8	9	9	10	11	11

Index date	6/5/2022	7/5/2022	8/5/2022	9/5/2022	10/5/2022	11/5/2022	12/5/2022	1/5/2023	2/5/2023	3/5/2023	4/5/2023	5/5/2023
Number	4,491	4,495	4,403	4,416	4,414	4,499	4,464	4,372	4,316	4,308	4,225	4,173
Percent of cohort (%)	11	11	11	11	11	11	11	11	11	11	10	10
Prescribed SGLT2i (n)	516	553	579	607	634	660	668	691	699	750	798	862
Prescribed SGLT2i (%)	12	12	13	14	14	15	15	16	16	17	19	21

Index date	6/5/2023	7/5/2023	8/5/2023	9/5/2023	10/5/2023	11/5/2023	12/5/2023	1/5/2024	2/5/2024	3/5/2024	4/5/2024	5/5/2024
Number	4,128	4,123	4,135	4,160	4,134	4,161	4,482	4,378	4,386	4,369	4,307	4,271
Percent of cohort (%)	10	10	10	10	10	10	10	11	10	10	10	10
Prescribed SGLT2i (n)	928	992	1,030	1,075	1,086	1,138	1,267	1,284	1,320	1,350	1,349	1,381
Prescribed SGLT2i (%)	23	24	25	26	26	27	28	29	30	31	31	32

Supplemental Table 7. Cumulative percentage of patients with type 2 diabetes in the BPA group (last ACR > 300 mg/gm and eGFR ≥ 30 ml/min up to 5 years lookback) prescribed SGLT2-inhibitor within 1 year of index date. BPA recommending SGLT2-inhibitor in patients with ACR > 300 mg/gm and eGFR ≥ 30 ml/min was implemented in June 2022 and expanded to include patients with eGFR ≥ 60 ml/min in February 2023.

Index date	6/5/2021	7/5/2021	8/5/2021	9/5/2021	10/5/2021	11/5/2021	12/5/2021	1/5/2022	2/5/2022	3/5/2022	4/5/2022	5/5/2022
Number	1,684	1,677	1,680	1,679	1,680	1,716	1,714	1,695	1,691	1,705	1,714	1,712
Percent of cohort (%)	4	4	4	4	4	4	4	4	4	4	4	4
Prescribed SGLT2i (n)	112	128	133	138	148	162	177	180	185	208	217	232
Prescribed SGLT2i (%)	7	8	8	8	9	9	10	11	11	12	13	14

Index date	6/5/2022	7/5/2022	8/5/2022	9/5/2022	10/5/2022	11/5/2022	12/5/2022	1/5/2023	2/5/2023	3/5/2023	4/5/2023	5/5/2023
Number	1,767	1,781	1,801	1,829	1,846	1,910	1,921	1,913	1,916	1,949	1,931	1,943
Percent of cohort (%)	4	4	4	5	5	5	5	5	5	5	5	5
Prescribed SGLT2i (n)	239	255	273	288	314	339	342	362	375	420	456	516
Prescribed SGLT2i (%)	14	14	15	16	17	18	18	19	20	22	24	27

Index date	6/5/2023	7/5/2023	8/5/2023	9/5/2023	10/5/2023	11/5/2023	12/5/2023	1/5/2024	2/5/2024	3/5/2024	4/5/2024	5/5/2024
Number	1,927	1,920	1,946	1,953	1,959	1,974	2,060	2,024	2,022	2,028	2,027	2,018
Percent of cohort (%)	5	5	5	5	5	5	5	5	5	5	5	5
Prescribed SGLT2i (n)	557	611	648	686	708	745	797	824	846	869	859	863
Prescribed SGLT2i (%)	29	32	33	35	36	38	39	41	42	43	42	43

Supplemental Table 8. Multiple linear regression model for outcome of SGLT2-inhibitor use divided by BPA vs. non-BPA group pre and post CDS rollout.

Variable	Coefficient	95% CI	P value
Age (per 10 yrs)	-0.02	-0.02 to -0.010	<0.001
Gender (reference is female)	-0.03	-0.03 to -0.02	<0.001
Race (reference is white)	0.002	-0.0007 to -0.005	0.16
Congestive heart failure	0.12	0.11 to 0.13	<0.001
ASCVD	0.04	0.03 to 0.05	<0.001
BPA (reference is targeted alert group)	-0.004	-0.01 to 0.002	0.24
Pre/post CDS rollout (reference post intervention)	0.08	0.07 to 0.09	<0.001
BPA*pre/post CDS rollout	0.07	0.06 to 0.08	<0.001

Supplemental Table 9. Characteristics in patients with renal indication for ACEi or ARB one year after rollout of CDS tools divided by whether patient prescribed ACEi or ARB within past year

Variable	Prescribed ACEi/ARB (n=7,789)	Not prescribed ACEi/ARB (n=2,635)	P value
Age (mean)	64	59	< 0.001
Gender (% male)	58	52	< 0.001
Race (%)			< 0.001
White	73	65	
Black	4	5	
Asian	8	8	
Hispanic	9	13	
Other	6	9	
Hypertension (%)	82	53	< 0.001
Congestive heart failure (%)	7	8	0.3
ASCVD (%)	21	17	< 0.001
CKD (%)	32	26	< 0.001
eGFR (median, ml/min)	83	92	< 0.001
ACR (median, mg/gm)	93	81	< 0.001
Followed by nephrology (%)	11	8	< 0.001

Supplemental Table 10. Characteristics in patients with CKD and type 2 diabetes one year after rollout of CDS tools divided by whether patients were included in the BPA vs. non-BPA group

Variable	BPA target (n=1,718)	BPA non-targeted (n=2,455)	P value
Age (median)	62	73	< 0.001
Gender (% male)	61	43	< 0.001
Race (%)			
White	66	84	
Black	5	5	
Asian	10	5	
Hispanic	12	2	
Other	9	4	
Hypertension (%)	82	87	< 0.001
Congestive heart failure (%)	9	12	0.006
ASCVD (%)	22	29	< 0.001
eGFR (median, ml/min)	79	49	< 0.001
Followed by nephrology (%)	19	18	0.8

Supplemental Figure 1. Timeline of quality improvement interventions as well as snapshots of handouts given end of visit or mailed to patient, provider handouts with best practice recommendations, and BPA in electronic health records.

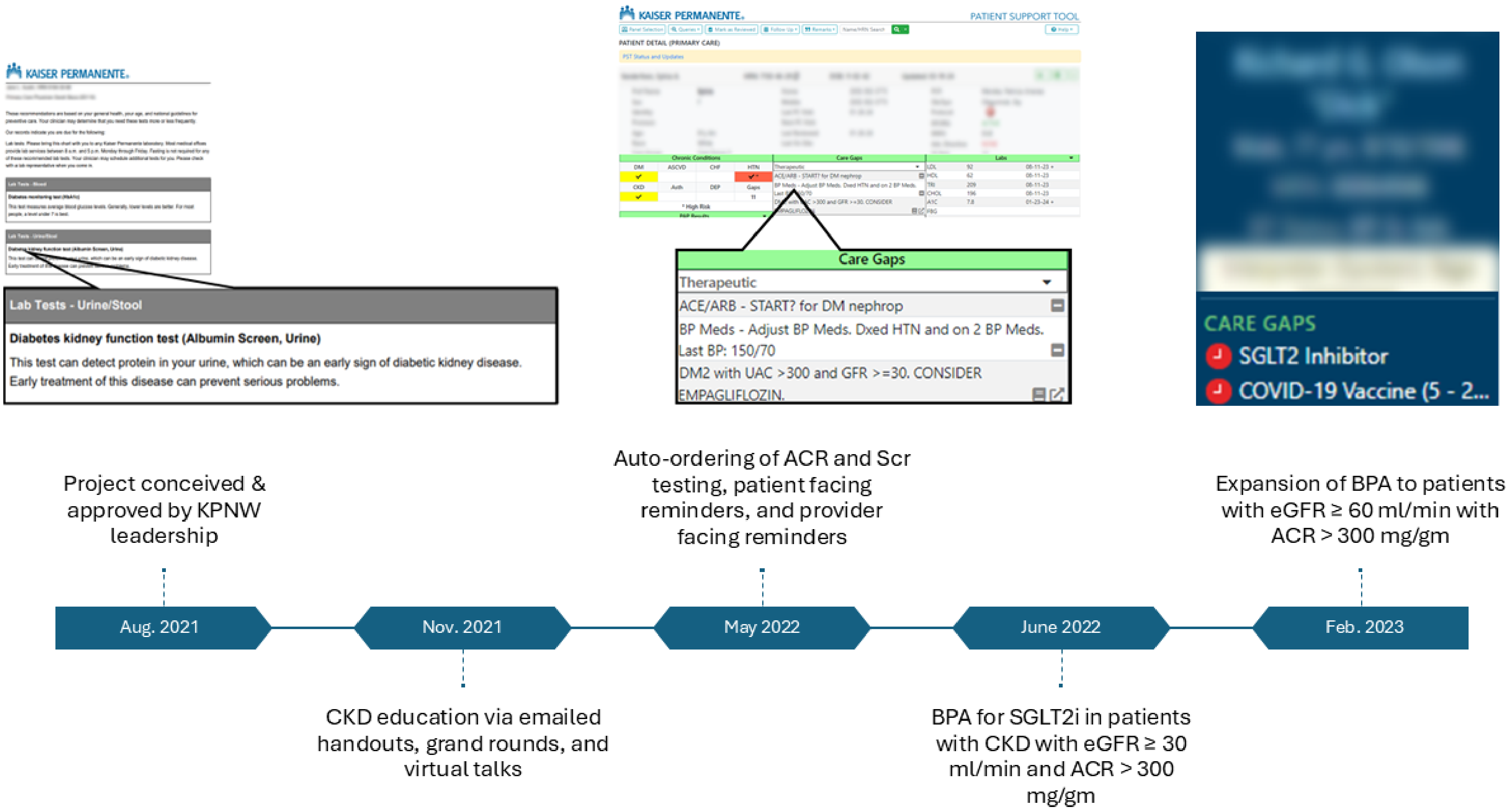

Supplemental Figure 2: Patients in BPA group (DM2 with ACR > 300 mg/gm and eGFR ≥ 30 ml/min) split by SGLT2-inhibitor use, not on SGLT2-inhibitor and firing the BPA, not firing the BPA due to missing ACR or eGFR, or not firing the BPA due to exclusions (history of DKA, kidney transplant, pregnant, history of Fournier's gangrene, diagnosis of foot or leg ulcer within 3 months, polycystic kidney disease, or missing CKD diagnosis). Percent of patients who were not firing the BPA due to exclusions decreased after February 2023 after exclusion for missing CKD diagnosis was removed.

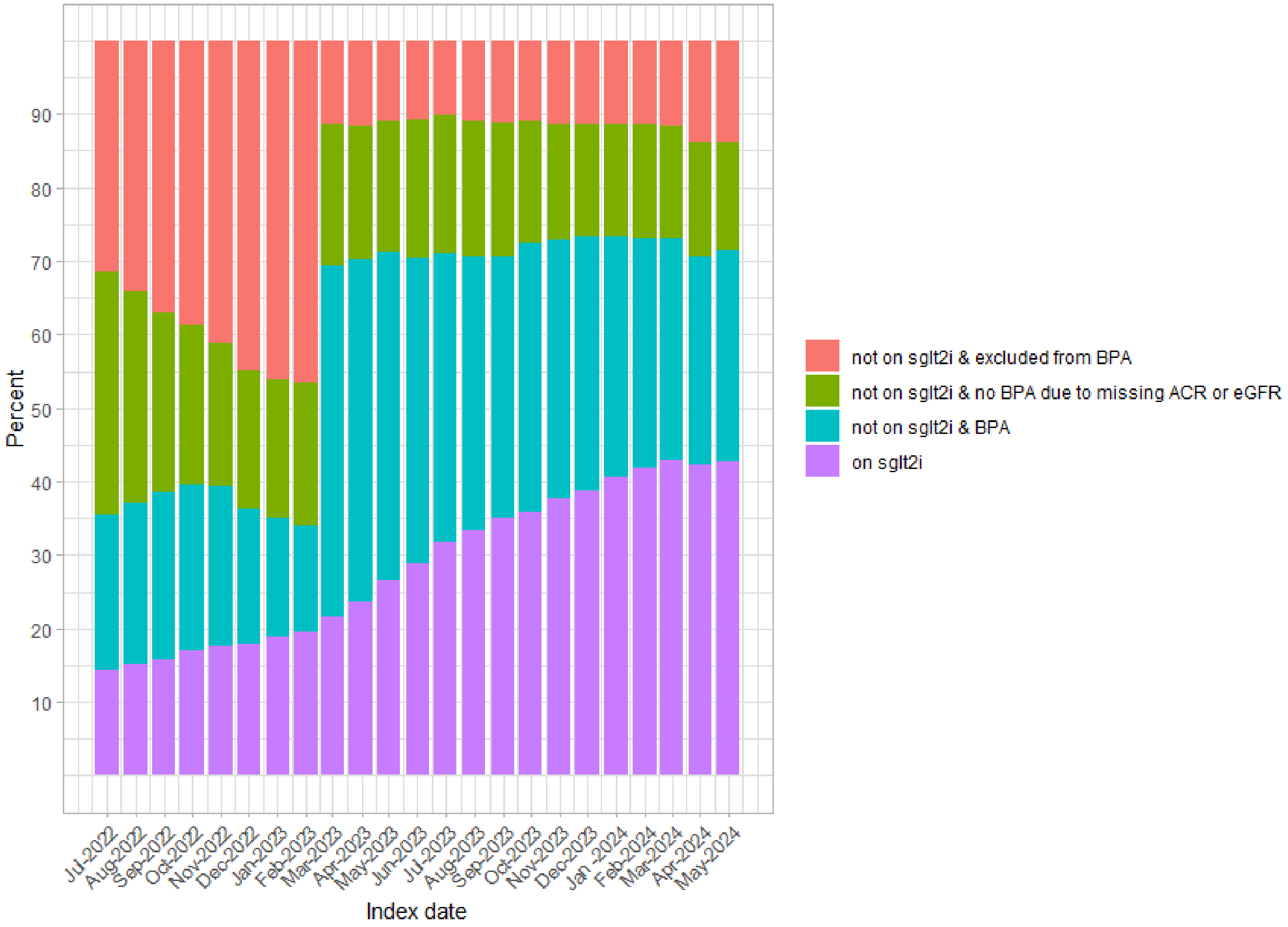

Supplemental Figure 3: Bar plot of patients with CKD with indication for nephrology co-management (most recent ACR ≥ 1200 mg/gm or eGFR < 30 ml/min with CKD up to 5 years lookback) with completed nephrology visit within 1 year.

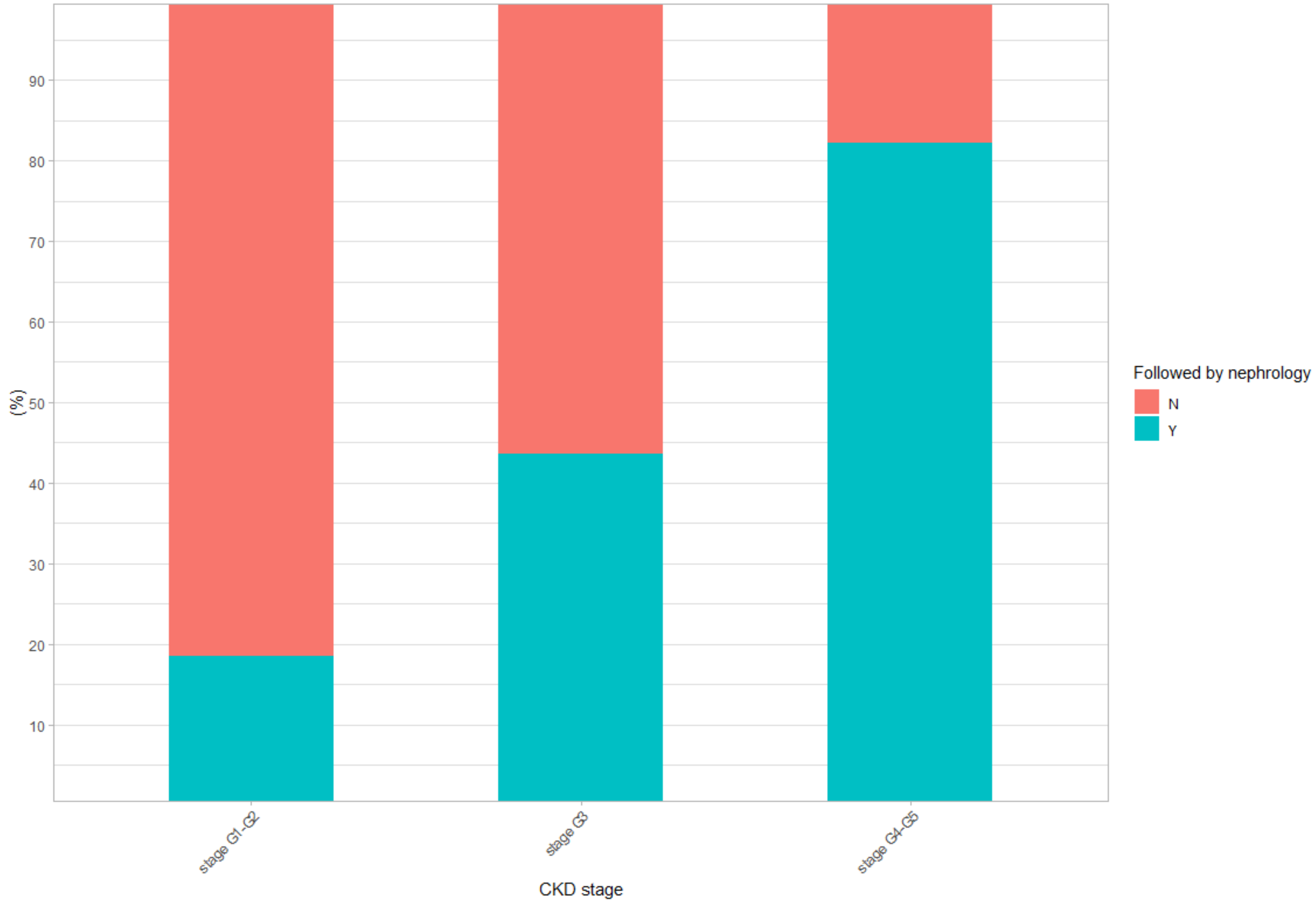

Supplemental Figure 4: A) P-chart of patients with diabetes with last hgba1c < 8%. B) P-chart of patients with diabetes with controlled blood pressure (defined as last blood pressure less than 140/90 mmHg). C) P-chart of patients with diabetes with retinal screening done within 1 year. Large increase seen in October 2022 in the retinal screening group was due to including encounters which had not been included due to incorrect coding. Points prior to October 2022 were excluded from calculating mean line for graph C. Implementation of auto-lab ordering, printed reminders to providers, and automated patient reminders occurred in May 2022 (vertical line) with the red center horizontal line representing the mean and the upper and lower dashed horizontal lines representing 3 standard deviations from the mean.

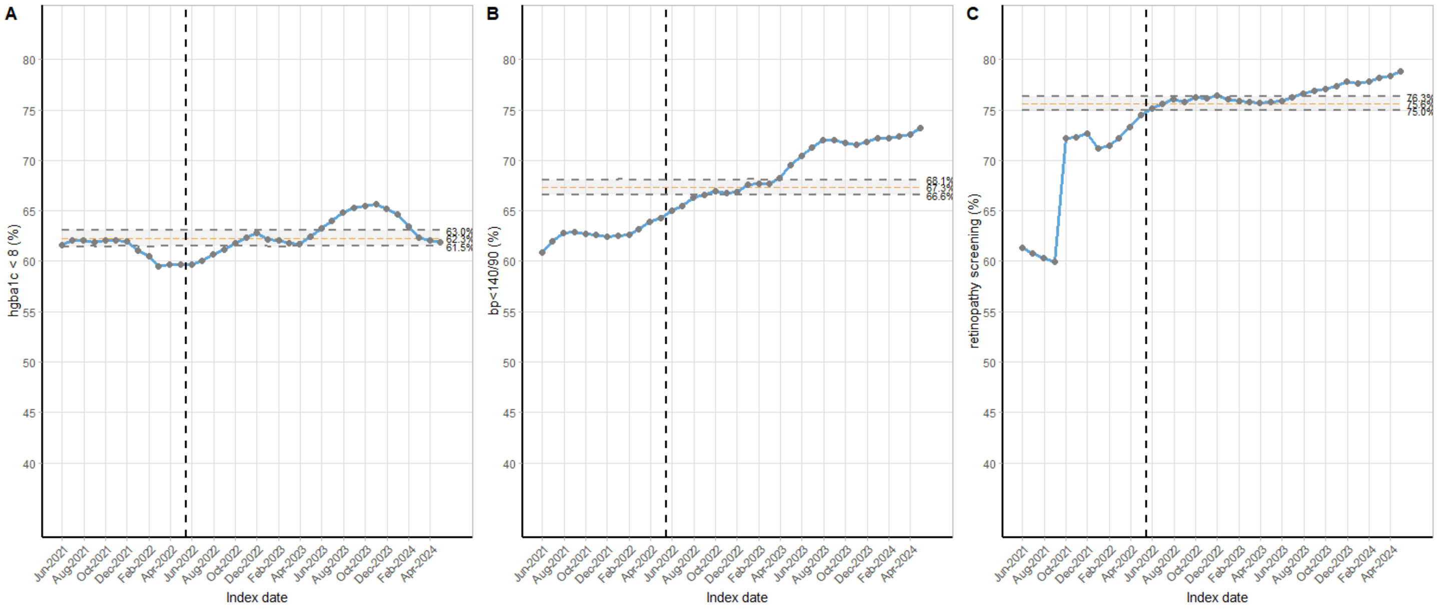

Supplement: Supplementary file 2 [file kidney360-6-01501-s002.pdf]
